# Intersectional inequalities in depressive symptoms according to gender, migration, and education in 30 European countries

**DOI:** 10.1186/s12889-026-28473-z

**Published:** 2026-07-10

**Authors:** Olaf von dem Knesebeck, Daniel Lüdecke, Nico Vonneilich

**Affiliations:** Institute of Medical Sociology, University Medical Center Hamburg Eppendorf, Martinistr. 52, Hamburg, 20246 Germany

**Keywords:** Depressive symptoms, Europe, Gender, Education, Migration, Intersectional inequalities

## Abstract

**Background:**

Intersectional approaches offer the opportunity to analyse the interplay of different social factors and reach a deeper understanding of inequalities in depressive symptoms. There is a need for further research on intersectional inequalities in depressive symptoms as the few previous studies focussed on individual countries. Therefore, we aimed to explore intersectional inequalities in depressive symptoms according to gender, migration, and education in 30 European countries.

**Methods:**

Analyses made use of the recent wave (round 11) of the cross-sectional European Social Survey (ESS, 2023/2024). Samples of the ESS are supposed to be representative of all persons aged 15 years and over resident within private households in each country. Individuals are selected by strict random probability methods at every stage. Overall sample size was *N* = 50,116. Depressive symptoms were assessed by the CES-D-8. Intersectional multilevel analysis of individual heterogeneity and discriminatory accuracy (MAIHDA) was conducted.

**Results:**

Women and respondents with a low education reported significantly more depressive symptoms in most European countries. People with a migration background were more affected than those without, although differences were significant in a few countries only. Social inequalities in depressive symptoms were attributable to additive effects of education, gender, and migration, with gender and education contributing most to these inequalities. Analyses across 12 intersectional strata (combining subgroups of education, gender, and migration) showed that the differences between the most (women with a migration background and low education) and least affected strata (men with high education and no migration background) were significant.

**Conclusions:**

The study revealed additive social inequalities in depressive symptoms according to education, gender, and migration in 30 European countries. Results indicate the potential of MAIHDA to identify vulnerable groups affected by multiple disadvantages that can be addressed in interventions to reduce social inequalities in depression.

**Supplementary Information:**

The online version contains supplementary material available at 10.1186/s12889-026-28473-z.

## Background

Social determinants of mental health comprise different structural and cultural conditions to which people are exposed to in their lives (e.g. economic stability, access to education and health care). These conditions “[…] are shaped by the distribution of money, power and resources at global, national and local levels, which are themselves influenced by policy choices” [1]. They affect mental disorders like depression and contribute to inequalities within and between populations [2]. In this regard, education is an important structural condition and it is well known that there are educational inequalities in depressive symptoms, i.e. the lower the educational attainment, the greater the risk of experiencing depressive symptoms [3]. Studies demonstrated that this pattern can be observed in many European countries [4, 5], although there were country differences in the magnitude of these inequalities [4, 6]. Large inequalities were found in countries like Hungary, while smaller inequalities emerged in Scandinavian countries [4, 7]. Among the components explaining the educational gradient in depressive symptoms are poorer coping mechanisms, stress exposure, behavioural risk factors, fewer material resources, and restricted access to health care for people with lower education [3, 8]. Studies on educational inequalities in depressive symptoms have been criticized for disregarding within-group heterogeneity and concentrating on single-axis inequalities [9, 10]. They did not consider combinations of or interactions with other social inequality indicators that have been found to be social determinants of depressive symptoms such as gender, migration background, or ethnicity.

In terms of gender, it is well established that women are about twice as likely to report depressive symptoms and develop a depression during their lifetime [11–13]. According to a meta-analysis, these gender differences haver their peak in adolescence, then decline and remain stable during adulthood [14]. Biological, psychological, and environmental risk factors contribute to the gender gap in depressive symptoms [13]. Among the latter are stress exposure, violence against girls and women, and structural gender inequalities which describe the extent to which women and men have unequal opportunities to access the scarce and valued resources of their society [12]. European studies indicate that people with a migration background as well as ethnic minorities more often report depressive symptoms than people who do not have a migration background or do not belong to ethnic minorities [15–17]. However, there were variations between migrant generations and across countries. There is evidence showing a mental health advantage for people with lower age at migration and a mental health decline over years after immigration [18]. Health selection in the countries of origin, especially in the context of labour migration, and selection during the process of migration itself can contribute to the explanation of health advantages at the time of arrival [19, 20]. Socioeconomic disadvantages, discrimination, less access to health services (e.g. due to language barriers) and increased health risks at the workplace and elsewhere can explain the deterioration of early health advantages over the years after migration [16, 21–25]. Studies on gender and migration related inequalities in depression also lacked an intersectional perspective.

The theoretical concept of intersectionality has its roots in Black feminist activist thought, notably the Combahee River Collective [26] and was developed to understand combinations of multiple social categories (e.g. race and gender) leading to disadvantage, discrimination, and inequality [27]. The experiences of Black women being subjected to multiple forms of inequality and discrimination led to the analysis of interlocking systems of privilege and disadvantage. According to this concept, these women occupy a unique social position that is not fully understood when racism and sexism were treated as separate issues. Thus, individuals located at the intersection of multiple systems of power and stratification have identities and positions that shape their experiences in society and are entirely distinct from the sum of the social categories. Intersectionality emphasises the multidimensional aspects of inequalities and underlines their potential effects, especially when different forms of disadvantage are experienced simultaneously [28–31]. In this regard, social categories (like education, gender, and migration) can have additive or multiplicative statistical effects. While the former indicate that the sum of privileges and/or disadvantages operates relatively uniformly across different social groups, the latter imply that specific combinations create unique, interlocking vulnerabilities [31]. It is important to consider, that equating intersectionality with multiplicative effects risks oversimplification, since intersectionality also concerns power, history, and structures shaping inequalities [32]. The Intersectional Multilevel Analysis of Individual Heterogeneity and Discriminatory Accuracy (MAIHDA) approach has been developed to study intersectionality [33, 34]. It is a tool for integrating the intersectional framework into quantitative study designs. In line with the theoretical concept, this tool provides the opportunity to model intersectional strata as social positions that may exhibit unique patterns of inequality that cannot be reduced to the main effects of single social categories.

There are a few studies analysing intersectional inequalities in depressive symptoms using the MAIHDA approach. A German study revealed an educational gradient in depressive symptoms, with differences within each educational group when gender and migration background were considered [35]. Moreover, it was shown that the differences between the social strata were largely explained by additive effects. In another study from Germany, significant interactions between four social categories (gender, lesbian/gay/bisexual/transgender (LGBT) status, migration, and education) for depressive symptoms were shown [36]. Evans and Erickson [10] examined intersectionality among adolescents and young adults in the U.S. along the dimensions of gender, race/ethnicity, immigration status, and family income. They found inequalities between the social strata, with women, racial/ethnic minorities, immigrants, and low income strata reporting higher depression scores.

Overall, there is a need for further research on intersectional inequalities in depressive symptoms as the few existing studies focussed on individual countries. By applying the MAIHDA approach, we explored intersectional inequalities in depressive symptoms according to gender, migration background, and education in 30 European countries, based on a current dataset (2023/2024). More specifically, the following research questions were investigated: (1) What is the magnitude of social inequalities in depressive symptoms according to gender, migration background, and education in 30 European countries? (2) Are the inequalities additive or multiplicative? (3) Which of the three social categories contributes most to the inequalities? (4) Do the intersectional strata (combining subgroups of education, gender, and migration) significantly differ regarding depressive symptoms?

## Methods

### Study design

Analyses made use of round 11 of the European Social Survey (ESS, 2023/2024 [37]). The ESS is a cross-national survey with rigorous methodology. Every two years, face-to-face interviews are conducted with newly selected, cross-sectional samples [38]. The survey assesses attitudes, beliefs and behaviour patterns of diverse populations in a number of European countries. In the present analyses, 30 countries were included (Austria, Belgium, Bulgaria, Croatia, Cyprus, Estonia, Finland, France, Germany, Greece, Hungary, Iceland, Ireland, Israel (as a non-European country), Italy, Latvia, Lithuania, Montenegro, the Netherlands, Norway, Poland, Portugal, Serbia, Slovak Republic, Slovenia, Spain, Sweden, Switzerland, Ukraine, and United Kingdom (UK)).

### Sample

Samples of the ESS are (supposed to be) representative of all persons aged 15 years and over resident within private households in each country [39]. Individuals are selected by strict random probability methods at every stage. For the present analyses of ESS (round 11), overall sample size was *N* = 50,116, with the smallest size in Cyprus (*n* = 685) and the largest in Italy (*n* = 2,865). Sample sizes for each country are reported in Table 1. Average response rate across all 30 countries was 41%, with a range from 23.7% in Sweden and Israel to 76.6% in Bulgaria [37, 40].

**Table 1:** Sample characteristics (European Social Survey, round 11, 2023/2024)

Country	*N*	Women (%)	Migration, yes (%)	Education, low (%)	CES-D-8* (mean; SD)
Austria	2,354	57.8	16.3	15.1	4.9; 3.6
Belgium	1,594	49.2	23.6	18.3	5.3; 4.0
Bulgaria	2,239	52.2	2.0	18.2	5.9;4.6
Croatia	1,563	54.5	20.8	21.5	5.4; 4.0
Cyprus	685	54.9	14.7	26.8	6.4; 4.5
Estonia	1,293	55.4	28.2	12.5	6.1; 3.9
Finland	1,563	50.7	7.3	16.2	4.8;3.2
France	1,771	50.6	21.8	18.0	4.9; 4.0
Germany	2,420	49.8	25.4	9.4	5.6; 3.7
Greece	2,757	55.1	5.0	24.3	7.0; 3.7
Hungary	2,118	60.6	4.7	19.1	6.5; 4.3
Iceland	842	50.4	17.3	21.7	5.1; 3.9
Ireland	2,017	55.1	19.9	28.6	4.5; 3.7
Israel	906	51.4	50.3	9.2	7.3; 4.3
Italy	2,865	53.3	9.7	42.2	6.1; 4.0
Latvia	1,252	65.2	22.5	12.7	7.4; 4.2
Lithuania	1,365	61.5	12.7	14.3	6.8; 3.9
Montenegro	1,609	46.1	8.7	14.4	8.1; 4.9
Netherlands	1,695	50.3	18.2	26.2	4.6; 3.4
Norway	1,337	49.7	19.3	11.3	4.5; 3.2
Poland	1,442	53.2	3.3	30.6	5.0; 4.5
Portugal	1,373	57.9	17.0	48.9	6.9; 4.8
Serbia	1,563	53.2	16.0	16.9	6.1; 4.2
Slovak Rep.	1,442	53.5	4.0	7.8	6.5; 3.9
Slovenia	1,248	51.3	21.2	16.9	4.8; 3.7
Spain	1,844	52.5	19.2	45.4	5.7; 4.4
Sweden	1,230	47.8	23.1	12.0	5.0; 3.6
Switzerland	1,348	49.6	43.6	16.5	4.6;3.5
UK	1,684	51.1	26.3	32.5	5.9; 4.4
Ukraine	2,661	64.5	14.7	9.2	7.9; 5.0
*Total*	*50*,*116*	*53.9*	*16.6*	*21.1*	*5.9; 4.2*

* range 0–24

*SD* Standard deviation

### Measures

Depressive symptoms were measured using the Center for Epidemiologic Studies Depression Scale (CES-D-8) [41], which has also been applied in previous rounds of the ESS [37] and was found to be valid for European comparisons [12]. Participants completing the CES-D-8 indicated the frequency of various depressive symptoms they had experienced during the preceding week (felt depressed, felt that everything was an effort, slept badly, felt lonely, felt sad, could not get going, enjoyed life (reverse coding), or felt happy (reverse coding)). Response categories ranged from none or almost none of the time (0) to all or almost all of the time (3). The total score (ranging from 0 to 24) was derived by summing the responses to these eight items, with higher scores indicating a greater frequency of reported depressive symptoms.

Gender (woman/man, i.e. sex is used as a proxy for gender), migration background, and education were considered as indicators of social inequalities. Migration background was defined based on individuals’ country of birth and their parents’ country of birth [42]. Accordingly, two groups were defined: The group with migration background included individuals born in a foreign country or those with at least one foreign-born parent. The group without migration background consisted of individuals who themselves and their parents were born in the country of residence. This definition was adopted to ensure consistency with previous research utilizing ESS data [16]. Education was measured using the ISCED 2011 scale [43] and recoded into three categories: low (primary and lower secondary education, ISCED 0–2), medium (upper secondary and post-secondary non-tertiary education, ISCED 3–4), and high (tertiary education, ISCED 5–8). Age was used as a covariate because it is a fundamental demographic factor associated with depressive symptoms.

### Analyses

Descriptive statistics for the 30 countries and for the pooled sample are reported using proportions for categorical, and means and standard deviations for numeric variables. Estimated marginal means were calculated for the CES-D-8 score by using simple linear models with interactions between country and one of the three inequality indicators (gender, education and migration background). Pairwise comparisons were used to calculate p-values for the differences between categories of the inequality indicators.

We investigated intersectional inequalities using the MAIHDA approach for the pooled sample. MAIHDA employs multilevel models where social strata are treated as higher-level random effects [33, 34]. By treating group indicators (in this case, intersectional strata) as random effects, these models perform ‘partial pooling’, acting as a statistical compromise between completely ignoring group differences and fitting separate models for each group. This approach accounts for the clustered nature of the data and produces stable estimates, even when intersectional groups have very few observations [44, 45]. In this study, we used linear mixed effects models, controlling for individual-level age as a fixed effect. The initial MAIHDA step involved calculating the Intraclass Correlation Coefficient (ICC) in a baseline model to quantify the overall inequality attributable to social strata (formulas of the models are shown in the Supplement). Subsequently, we ran models incorporating each of the three indicators (gender, migration background, and education) as additional fixed effect to determine their individual contribution to inequalities, measured by the Proportional Change in Variance (PCV). A larger PCV indicates a greater contribution. Finally, a full model including all three indicators as individual-level fixed effects was estimated to assess additive or multiplicative effects on inequalities. A high PCV (close to one) suggests that the (additive) fixed effects absorb all higher-level variance, meaning we can assume additive effects regarding the intersectional strata. A considerably lower PCV points to interaction effects between the strata. We report regression coefficients and their 95% confidence intervals. The CES-D-8 scores for the 12 social strata (combining subgroups of education (three groups), gender (two groups), and migration (two groups)) were visualized using ranked predictions (estimated marginal means), illustrating a ranking of strata based on depressive symptoms. Differences between ranked predictions were tested for statistical significance applying pairwise comparisons with Holm correction for multiple testing [46]. To account for unobserved country-level heterogeneity, all models included the country of residence as an additional random intercept. Country and intersectional strata were modelled as cross-classified random effects. Furthermore, analysis weights were used in regression models [47]. All analyses were performed using R and the glmmTMB, performance, and model based packages [48, 49].

## Results

Mean age of the respondents in the pooled sample was 51.6 years, with a range from 43.3 years in Iceland to 56.6 years in Latvia (data not shown). As shown in Table 1 and 53.9% of the overall sample were women (range 46.1% to 65.2%). Overall, 16.6% of the respondents had a migration background, with a considerable range from 2.0% (Hungary) to 50.3% (Israel). Depressive symptoms were most pronounced in Montenegro (mean 8.1), while Ireland and Norway showed the lowest values (mean 4.5).

Women reported significantly more depressive symptoms than men in all included European countries (*p* < 0.05), except Latvia, Ireland, and Iceland (Table 2). Overall, people with a migration background showed more depressive symptoms than those without, although differences were not significant in most countries. These differences emerged to be significant in Belgium, Estonia, Germany, Iceland, the Netherlands, Norway, Sweden, Switzerland, and Ukraine, while in Cyprus, Israel, and Montenegro respondents without a migration background reported significantly more depressive symptoms than migrants. Depressive symptoms were significantly more pronounced among respondents with low education compared to those with high education in most countries (exceptions were Cyprus, Estonia, Finland, Iceland, Ireland, Latvia, Montenegro, Slovenia, Sweden, and Switzerland).

**Table 2:** Depressive symptoms (CES-D-8, mean) according to gender, migration background, and education (European Social Survey, round 11, 2023/2024)

Country	Women; men	p*	Migration yes; no	p*	Education low; high	p*
Austria	5.2; 4.6	< 0.001	5.0; 4.9	0.554	5.5; 4.5	0.013
Belgium	5.8; 4.7	< 0.001	6.0; 5.0	< 0.001	6.5; 4.6	< 0.001
Bulgaria	6.3; 5.5	< 0.001	6.2; 5.9	0.658	7.3; 5.0	< 0.001
Croatia	5.9; 4.7	< 0.001	5.4; 5.4	0.999	6.8; 4.0	< 0.001
Cyprus	7.3; 5.3	< 0.001	5.5; 6.5	0.020	6.7; 5.2	0.225
Estonia	6.5; 5.6	< 0.001	6.7; 5.8	< 0.001	6.2; 5.8	0.228
Finland	5.1; 4.6	0.013	4.7; 4.9	0.710	5.1, 4.7	0.529
France	5.6; 4.2	< 0.001	5.1; 4.8	0.150	5.5; 4.5	< 0.001
Germany	6.0; 5.2	< 0.001	6.0; 5.4	< 0.001	7.0; 5.0	< 0.001
Greece	7.4; 6.5	< 0.001	7.0, 7.0	0.947	7.8; 6.4	< 0.001
Hungary	6.8; 6.1	< 0.001	7.1; 6.5	0.095	7.9; 4.7	< 0.001
Iceland	5.1; 5.0	0.761	6.1; 4.8	< 0.001	6.6; 4.7	0.357
Ireland	4.7; 4.3	0.076	4.7; 4.5	0.346	4.6; 3.9	0.155
Israel	7.7; 6.6	< 0.001	7.0; 7.6	0.021	9.6; 6.8	< 0.001
Italy	6.7; 5.5	< 0.001	6.0; 6.1	0.488	7.3, 4.8	0.001
Latvia	7.5; 7.3	0.638	7.3; 7.4	0.575	7.1; 6.5	0.599
Lithuania	7.0; 6.5	0.023	7.0; 6.8	0.519	7.3; 5.7	0.079
Montenegro	8.3; 7.9	0.035	7.2; 8.2	0.008	9.9; 6.6	0.086
Netherlands	5.0; 4.1	< 0.001	5.4; 4.4	< 0.001	5.2; 4.3	< 0.001
Norway	4.9; 4.2	0.001	5.4; 4.3	< 0.001	5.5; 4.5	0.032
Poland	5.5; 4.5	< 0.001	5.0; 5.0	0.945	5.7; 4.6	< 0.001
Portugal	7.7; 5.7	< 0.001	6.6; 6.9	0.220	7.2; 5.6	< 0.001
Serbia	6.7; 5.5	< 0.001	6.3; 6.1	0.505	7.0; 5.3	0.004
Slovak Rep.	6.9; 5.9	< 0.001	6.8; 6.5	0.567	6.6; 5.0	0.013
Slovenia	5.3; 4.3	< 0.001	4.8; 4.9	0.863	5.9; 4.3	0.090
Spain	6.3; 5.0	< 0.001	5.9; 5.6	0.244	6.2; 5.0	< 0.001
Sweden	5.4; 4.7	0.002	5.8; 4.8	< 0.001	5.6; 5.0	0.148
Switzerland	5.0; 4.2	< 0.001	4.9; 4.3	0.007	5.1, 4.4	0.096
UK	6.3; 5.5	0.001	5.6; 5.9	0.147	6.5; 5.1	< 0.001
Ukraine	8.5; 6.9	< 0.001	9.0; 7.7	< 0.001	8.2; 6.6	< 0.001
*Total*	*6.3; 5.4*	*< 0.001*	*6.1; 5.8*	*< 0.001*	*6.7; 5.1*	*< 0.001*

* significance of pairwise comparison of estimated marginal means

The intersectional MAIHDA models for the overall pooled sample (including all countries) are shown in Table 3. ICC for model 1 was 8.6% indicating the proportion of variance in depressive symptoms attributable to the intersectional strata. After including migration in model 2, the ICC hardly decreased. Migration was not significantly associated with depressive symptoms in model 2. In model 3, in which gender was included, the ICC decreased to 7.0%, with women showing significantly higher values. We found the highest PCV (47.9%) in model 4, when education was included as main effect. Respondents with low education showed significantly higher values of depressive symptoms. Finally, the high value of PCV (which is close to one) in model 5 indicated that the intersectional inequalities found are attributable to additive effects of gender, migration background, and education. Depressive symptoms were significantly increased among people with a migration background, women, and respondents with a low and medium education in the fully adjusted model.

**Table 3:** Gender, migration background, education, and depressive symptoms (CES-D-8), MAIHDA regression models, adjusted for age; European Social Survey, round 11, 2023/2024, weighted pooled sample (*N* = 49,062)

	Model 1 Estimate (95%-CI)	Model 2 Estimate (95%-CI)	Model 3 Estimate (95%-CI)	Model 4 Estimate (95%-CI)	Model 5 Estimate (95%-CI)
Intercept	5.15 (4.55, 5.75)	4.93 (4.18, 5.68)	4.60 (3.96, 5.24)	4.43 (3.72, 5.14)	3.67 (3.24, 4.10)
Age	0.02 (0.01, 0.02)	0.02 (0.01, 0.02)	0.02 (0.01, 0.02)	0.02 (0.01, 0.02)	0.02 (0.01, 0.02)
Migration (yes)		0.44 (-0.52, 1.40)			0.44 (0.20, 0.69)
Gender (woman)			1.09 (0.32, 1.86)		1.08 (0.84, 1.33)
Education (low)				1.46 (0.57, 2.34)	1.46 (1.15, 1.76)
Education (medium)				0.69 (-0.19, 1.57)	0.67 (0.37, 0.98)
ICC	0.086	0.083	0.070	0.067	0.046
PCV	-	0.066	0.404	0.479	0.949

*CI* Confidence Interval, *ICC* Intraclass Correlation Coefficient, *PCV* Proportional Change in Variance

Reference categories: migration (no), man, high education

**Fig. 1:**
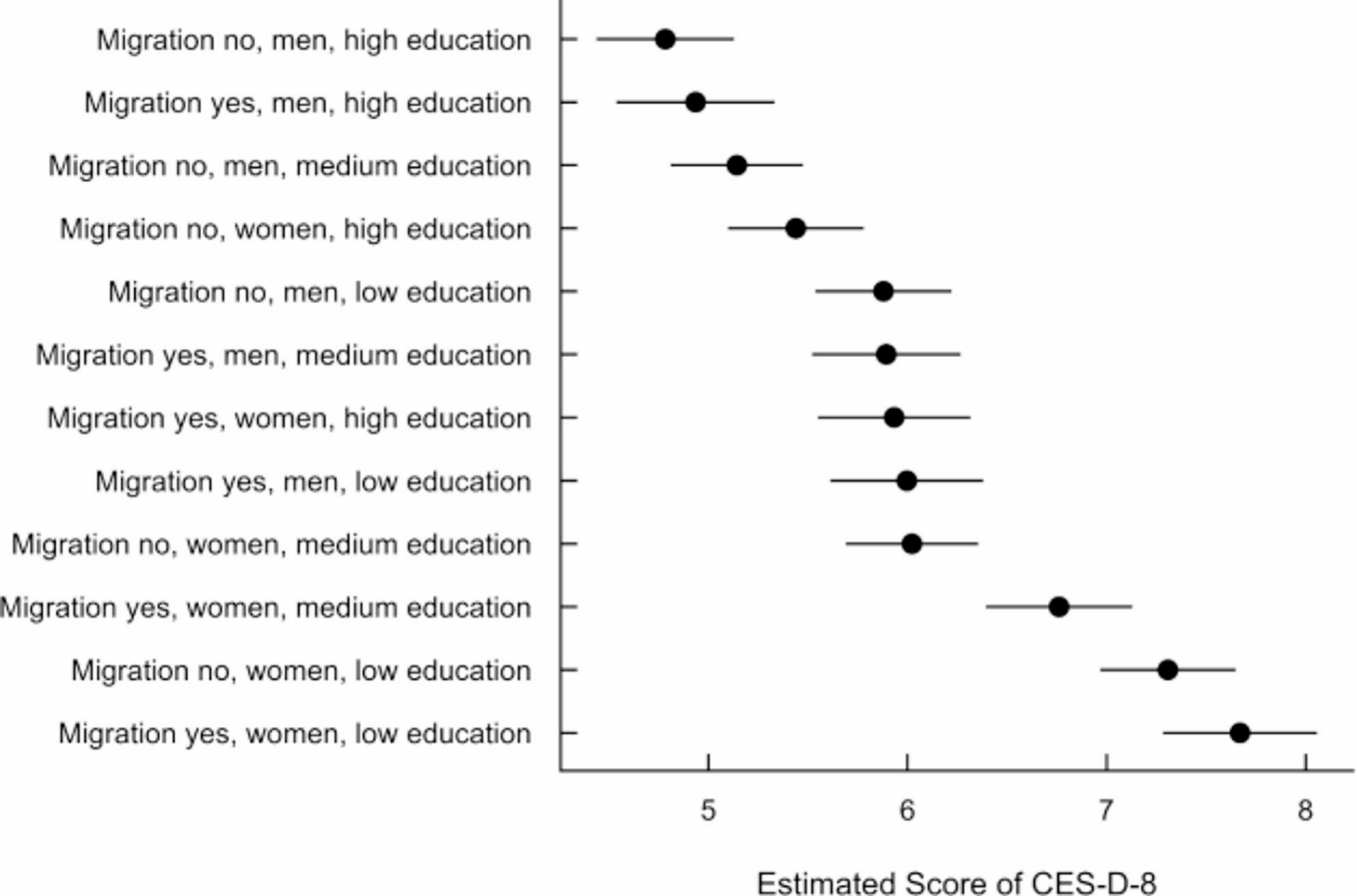
Estimated marginal means of depressive symptoms (CES-D-8) across 12 intersectional strata defined by combinations of gender (men/women), migration background (yes/no), and education (low/medium/high) based on MAIHDA regression model (*N* = 49,062)

Figure 1 presents the estimated marginal means (emm) of depressive symptoms across the 12 intersectional strata for the pooled sample. Men with high education and no migration background reported least depressive symptoms (emm = 4.78), while women with a migration background and low education were most affected by depressive symptoms (emm = 7.67). The differences between the most and least deprived intersectional strata were statistically significant (*p* < 0.001).

## Discussion

### Summary and interpretation

Based on a recent cross-sectional survey in 30 European countries and by applying MAIHDA [33, 34], we explored intersectional inequalities in depressive symptoms according to education, gender, and migration. The underlying goal was to overcome the traditional approach of studies on social variations in depression which usually focus on a single dimension of inequality. Analyses indicated significant associations of depressive symptoms with female gender and low education while associations with migration were less pronounced. Social inequalities in depressive symptoms were attributable to additive (and not multiplicative) effects of education, gender, and migration, with gender and education contributing most to these inequalities. Analyses across 12 intersectional strata (combining subgroups of education (three groups), gender (two groups), and migration (two groups)) showed that the differences between the most (women with a migration background and low education) and least affected strata (men with high education and no migration background) were statistically significant, although the differences of the estimated marginal means were relatively small. Among the three strata with the highest scores of depressive symptoms, there was none comprising men or respondents with high education. In contrast, among the three strata with the lowest scores there was none comprising women or respondents with low education. Nevertheless, no clear educational gradient emerged among the intersectional strata.

There are a few previous studies analysing intersectional inequalities in depressive symptoms using the MAIHDA approach. A recent German study also examined inequalities according to education, gender, and migration background [35] and found that education contributed most to the variance explained by the MAIHDA models. Like in our study, highly educated men with no migration background reported least and women with migration background with low education disclosed most depressive symptoms. Moreover, similar to our findings, the between-strata differences were largely explained by additive effects. This is in line with a U.S. study among adolescents and young adults [10] which found that intersectional inequalities in depression along the dimensions of gender, race/ethnicity, immigration status, and family income were mainly due to additive effects. Additive effects indicate that the direction and magnitude of the effects is the same for each social category considered [10]. For the results presented here, this would for example mean that having a low education has an adverse impact on depressive symptoms across the social strata of gender and migration in the European countries under study.

Significantly increased depressive symptoms among low educated respondents were found in most of the included 30 European countries. Like in previous studies [4, 7], educational inequalities were less pronounced in Scandinavian countries (Finland, Iceland, and Sweden among others). It can be assumed that the comparably high welfare standards, egalitarian living conditions, and the implementation of preventive measures for mental health across many sectors in these countries reduce the impact of education [7]. Low education was significantly associated with more depressive symptoms in the pooled MAIHDA models. Increased symptom load among individuals with low education can be explained by a higher prevalence of different risk factors of depression like psychosocial stressors, adverse behaviours, and less access to health care [50, 51]. Moreover, a study from Germany showed that people with a low education have a lack of psychosocial resources like sense of control and resilience [8]. This lack of resources together with daily hassles mediated the association between education and depressive symptoms.

Women reported significantly more depressive symptoms in all included European countries, except Latvia, Ireland, and Iceland. Gender difference in depression symptoms was also small in Ireland and Iceland in previous European studies [11, 12]. Country differences in the gender gap of depressive symptoms can partly be explained by the magnitude of gender inequalities [12]. In countries where women have equal opportunities to access resources, the gender gap in depressive symptoms appears to be reduced. However, overall, this study confirms that women report significantly more symptoms than men. The MAIHDA models (PCV) indicated that gender contributed slightly less to the inequalities under study than education (please see models 3 and 4 in Table 3). Furthermore, intersectional analyses revealed that especially women with lower education were affected by depressive symptoms. In addition, among those with a low education, it makes a difference whether the person is a woman or a man.

Overall, people with a migration background showed more depressive symptoms than those without, although differences were not significant in most European countries. Significantly increased symptoms among people with no migration background were found in nine countries (Belgium, Estonia, Germany, Iceland, the Netherlands, Norway, Sweden, Switzerland, and Ukraine). In five of these countries, people with a migration background already reported significantly higher levels of depressive symptoms in 2014 [16] (exceptions were Belgium and Estonia, while Iceland and Ukraine were not included) and in 2006 [15] (exceptions were Belgium, Germany, and Ukraine, while Iceland was not included). It is likely that different factors like socioeconomic disadvantages, experiences of discrimination, less access to health services, and increased mental health risks contribute to the elevated symptoms of depression among people with a migration background in some countries [15, 16, 21, 24]. In the pooled MAIHDA-models, migration did not contribute much to the inequalities in depressive symptoms.

### Limitations

Although the ESS has high methodological standards [37], some limitations have to be considered when interpreting and evaluating the presented findings. First, the cross-sectional structure of the ESS data precludes causal conclusions. Second, non-response and cross-national differences in response rates may affect the results. Average response rate across all 30 countries was 41%, with a range from 23.7% in Sweden and Israel to 76.6% in Bulgaria. Although data was weighted to reduce the impact of low response rates, selection bias due to non-response cannot be ruled out. As response rates are expected to be lower in less healthy people and low educated groups, non-response might have led to an underestimation of some of the associations presented. Third, the ESS sample is likely to be not adequately representative for groups with a migration background as data collection was carried out in the respective national language(s). This probably led to an underrepresentation of marginalized groups. Moreover, we only differentiated two migration groups: One group included individuals born in a foreign country or those with at least one foreign-born parent. The other group consisted of individuals who themselves and their parents were born in the country of residence. While this definition of migration groups was adopted to ensure consistency with previous research utilizing ESS data [16], it in a way is crude because it, for example, does not distinguish between 1st and 2nd generation migrants. The small proportion of respondents with migration background in some included European countries (please see Table 1) hampered further differentiation. It can be expected that within populations with a migration background there is considerable variation regarding depressive symptoms, for example between European and Non-European migrants, asylum-seekers and non-asylums seekers, refugees and non-refugees and regarding the length of stay in host-countries [18]. Overall, our measure of migration background may underestimate related inequalities in depressive symptoms. Fourth, depressive symptoms were assessed by the eight-item version of the Center for Epidemiological Studies Depression Scale (CES-D scale) [41]. Although the measure was used in various waves of the ESS and the validity across gender, age groups, and European countries was confirmed [52, 53], it cannot be considered a clinical diagnostic tool. Finally, it must also be kept in mind that data in Ukraine was collected only in areas under the control of the government at the time of fieldwork and that data collection in Israel was also severely disrupted by the conflict there [37].

## Conclusions

To the best of our knowledge, this is the first study analysing intersectional social inequalities in depressive symptoms in a number of European countries. Intersectionality serves as a theoretical concept in social epidemiology to examine the effects of multiple forms of disadvantage or oppression. By applying the topical MAIHDA approach, we explored the social context of depression disparities with a focus on three key social determinants of mental health. The study revealed social inequalities in depressive symptoms according to education, gender, and migration in 30 European countries. Gender and education contributed more to these inequalities than migration. Women with a migration background and low education were most affected by depressive symptoms while men with high education and no migration background were least affected. Results indicate the potential of MAIHDA to identify vulnerable groups affected by multiple disadvantages that can be addressed in interventions to reduce social inequalities in depression. In this regard, the strategy of proportionate universalism seems promising [54, 55]. It refers to the access of universal services and interventions at a scale and intensity proportionate to the degree of the need of the social group. Thus, a combination of targeted and universal policies is needed to address health inequalities. For the findings presented, this means that targeted interventions for the identified vulnerable groups (e.g. for prevention and treatment of depressive symptoms) should be combined with structural interventions (e.g. promoting healthy environments or providing equal opportunities to access resources) to tackle social inequalities in depressive symptoms.

## Supplementary Information

Supplementary Material 1

## Data Availability

The dataset used is publicly available from https://www.europeansocialsurvey.org.

## Abbreviations

CES-D-8: Center for Epidemiologic Studies Depression Scale
CI: Confidence Interval
emm: estimated marginal means
ESS: European Social Survey
ICC: Intraclass Correlation Coefficient
ISCED: International Standard Classification of Education
MAIHDA: Multilevel Analysis of Individual Heterogeneity and Discriminatory Accuracy
PCV: Proportional Change in Variance
UK: United Kingdom
