# Supplementary material for: Intersectional inequalities in depressive symptoms according to gender, migration, and education in 30 European countries
Source: BMC Public Health. 2026 Jul 10;26:2116. doi: 10.1186/s12889-026-28473-z (PMC13352892; doi:10.1186/s12889-026-28473-z)
Supplement: Supplementary file 1 — Supplementary Material 1 [file 12889_2026_28473_MOESM1_ESM.docx]

**Formulas for the linear mixed effects models (cross-classified design) from the paper and their corresponding R implementation**

**Model 1**

$$y_{i}=\beta_{0}+\beta_{1}\left( {age}_{i} \right)+u_{j\left[ i \right]}+v_{k\left[ i \right]}+e_{i}$$

$$u_{j}\sim N(0,\sigma_{strata}^{2})$$

$$v_{k}\sim N(0,\sigma_{country}^{2})$$

$$e_{i}\sim N(0,\sigma_{e}^{2})$$

*Corresponding R Code:*

model <- glmmTMB(
 depression_score ~ age + (1 | strata) + (1 | country),
 weights = w,
 data = d
)

**Model 2**

$$y_{i}=\beta_{0}+\beta_{1}\left( {age}_{i} \right)+\beta_{2}\left( {migration}_{i} \right)+u_{j\left[ i \right]}+v_{k\left[ i \right]}+e_{i}$$

$$u_{j}\sim N(0,\sigma_{strata}^{2})$$

$$v_{k}\sim N(0,\sigma_{country}^{2})$$

$$e_{i}\sim N(0,\sigma_{e}^{2})$$

*Corresponding R Code:*

model <- glmmTMB(
 depression_score ~ age + migration + (1 | strata) + (1 | country),
 weights = w,
 data = d
)

**Model 3**

$$y_{i}=\beta_{0}+\beta_{1}\left( {age}_{i} \right)+\beta_{2}\left( {sex}_{i} \right)+u_{j\left[ i \right]}+v_{k\left[ i \right]}+e_{i}$$

$$u_{j}\sim N(0,\sigma_{strata}^{2})$$

$$v_{k}\sim N(0,\sigma_{country}^{2})$$

$$e_{i}\sim N(0,\sigma_{e}^{2})$$

*Corresponding R Code:*

model <- glmmTMB(
 depression_score ~ age + sex + (1 | strata) + (1 | country),
 weights = w,
 data = d
)

**Model 4**

$$y_{i}=\beta_{0}+\beta_{1}\left( {age}_{i} \right)+\beta_{2}\left( {education}_{i} \right)+u_{j\left[ i \right]}+v_{k\left[ i \right]}+e_{i}$$

$$u_{j}\sim N(0,\sigma_{strata}^{2})$$

$$v_{k}\sim N(0,\sigma_{country}^{2})$$

$$e_{i}\sim N(0,\sigma_{e}^{2})$$

*Corresponding R Code:*

model <- glmmTMB(
 depression_score ~ age + education + (1 | strata) + (1 | country),
 weights = w,
 data = d
)

**Model 5**

$$y_{i}=\beta_{0}+\beta_{1}\left( {age}_{i} \right)+\beta_{2}\left( {migration}_{i} \right)+\beta_{3}\left( {sex}_{i} \right)+\beta_{4}\left( {education}_{i} \right)+u_{j\left[ i \right]}+v_{k\left[ i \right]}+e_{i}$$

$$u_{j}\sim N(0,\sigma_{strata}^{2})$$

$$v_{k}\sim N(0,\sigma_{country}^{2})$$

$$e_{i}\sim N(0,\sigma_{e}^{2})$$

*Corresponding R Code:*

model <- glmmTMB(
 depression_score ~ age + sex + migration + education +
 (1 | strata) + (1 | country),
 weights = w,
 data = d
)
